# Does memantine show chemopreventive effect against mice 4T1 breast tumor model?

**DOI:** 10.55730/1300-0144.5381

**Published:** 2022-01-24

**Authors:** Gülşah ALBAYRAK, Elif Burcu BALİ, Funda DEMİRTAŞ KORKMAZ, Emin Ümit BAĞRIAÇIK

**Affiliations:** 1 Department of Medical Biology, Faculty of Medicine, Ufuk University, Ankara, Turkey; 2 Department of Medical Services and Techniques, Vocational School of Health Services, Gazi University, Ankara, Turkey; 3 Department of Medical Biology, Faculty of Medicine, Giresun University, Ankara, Turkey; 4 Department of Immunology, Faculty of Medicine, Gazi University, Ankara, Turkey

**Keywords:** 4T1, triple negative breast cancer, memantine, chemoprevention, DNA methylation

## Abstract

**Background/aim:**

Cancer cells express higher levels of N-methyl-d-aspartate (NMDA) receptor. In this study, we aimed to use memantine as a potential blocker to inhibit the action of the NMDA receptor in cancer cells in vivo in order to investigate the potential chemopreventive effect of memantine in 4T1 tumor-bearing mice.

**Materials and methods:**

To determine the potential chemopreventive effect of the compound, mice weights, tumor volumes, spleen IL-6, and tumor DNA methylation levels were investigated. A total of 26 Balb/c female mice were allocated into three groups. G1 (n = 6): tumor control group, G2 (n = 10): low dose (5mg/kg) memantine group, G3: high dose (10 mg/kg) memantine group (n = 10). G1 was inoculated with 4T1 cells without any memantine treatment. G2 and G3 were pretreated with 5 and 10 mg/kg memantine daily intraperitoneal (ip) injection (weekend off) for 10 days, respectively. Both G2 and G3 were subdivided into two groups as G2a (n = 4) and G3a (n = 4): tumor free groups and G2b (n = 6) and G3b (n = 6) tumor bearing groups.

**Results:**

Our results revealed that G3: high dose (10 mg/kg) memantine group, significantly (p = 0.0248) reduced the tumor volumes. We found that spleen IL-6 levels were significantly higher in memantine pretreated tumor free group p = 0.0204 ) We also found that high dose memantine treated tumor free group (G3a) has significantly lower genome-wide DNA methylation levels when compared to tumor control group (G1) p = 0.0012.

**Conclusion:**

To the best of our knowledge, it is the first study that highlights a potential chemopreventive effect of memantine in vivo in the mouse 4T1 breast tumor model. But further investigations should be carried out to explore the chemopreventive mechanism of action for memantine in cancer.

## 1. Introduction

Memantine is an N-methyl-d-aspartate (NMDA) receptor antagonist, which has been used since many years for the treatment of moderate-to-severe Alzheimer disease [1]. The N-methyl-d-aspartate receptors (NMDARs), also known as ionotropic glutamate receptors, are expressed by primary, recurrent, and aggressive human tumor cells [2,3]. Higher NMDAR/glutamate exporter expression correlates with poor prognosis in human cancers [4]. Glutamatergic signaling pathway is known to be dysregulated in Schizophrenia, Alzheimer’s disease, and brain tumors. Recently it has been found that this pathway is also dysregulated in other cancers such as melanoma, breast, and prostate carcinomas [5].

Repurposed drugs such as metformin have recently gained interest as anticancer agents in cancer treatment [6,7]. Randomized clinical trials are ongoing to investigate the effect of metformin in cancer chemoprevention [8]. Memantine as a remarkable repurposed drug has also been investigated since over the last decade in cancer therapy [2, 9–11].

In a Phase 3 trial, memantine is used as an adjuvant therapy to decrease side effects caused by whole-brain radiation therapy [12,13]. Memantine can also be safely used in combination with mefloquine, metformin and temozolomide as postradiation adjuvant therapy in newly diagnosed glioblastoma[14]. Ongoing research about repurposing memantine is not only limited to central nervous system tumors but also in other malignancies like lung carcinoma, acute leukemia, etc. [15,16]. In addition to these researches, it has been recently reported in vitro and in vivo anticancer effect of memantine on breast and lung carcinomas [17,18]. We have also wondered the in vivo protective effects of memantine on breast cancer cells. To the best of our knowledge, the in vivo chemopreventive activity of memantine has not been studied previously. Therefore, in the present study, we aimed to use memantine as a potential blocker to inhibit the action of NMDA receptor in cancer cells and investigate the potential in vivo chemopreventive effect in 4T1 tumor-bearing mice.

## 2. Materials and methods

### 2.1. Chemicals, cell culture, and animals

Memantine was provided from Sigma–Aldrich, St Louis, MO, USA. Memantine was dissolved in apyrogenic sterile distilled water. 4T1 mice breast cancer cell line was kindly gifted by Dr. Gunes Esendagli (Hacettepe University Cancer Institute, Ankara). Cells were grown in Roswell Park Memorial Institute (RPMI) 1640 medium with 10% Fetal bovine serum (South africa origin, Biowest, USA) in a 5% CO_2_ incubator at 37 ^o^C. Female Balb/c mice (6–8 weeks old, 18–22 g each) were purchased from Kobay Laboratory Animals Inc. (Ankara, Turkey). The mice were housed in the cages at 24 ± 1 °C, 12-h light-dark cycle with ad libitum access in Gazi University Laboratory Animals and Experimental Research Center, Ankara. This study was performed under Gazi University Animal Research Ethics Committee approval between March and May 2017 (Approval number G.U.ET-17.019).

### 2.2. In vivo experimental tumor model design

**Figure 1:**
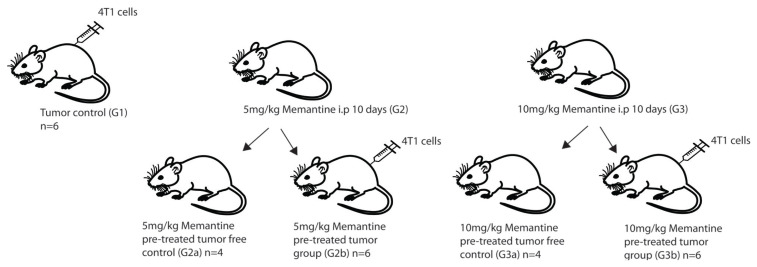
In vivo experimental tumor model design as groups representing G1 (n = 6): tumor control group, G2 (n = 10): low dose (5mg/kg) memantine group, G3 (n = 10): high dose (10 mg/kg) memantine group.

In this study, Balb/c allograft breast cancer model was used to determine chemopreventive effect of memantine in breast cancer. Dose calibration of memantine (5 and 10 mg/kg/day) was determined according to the previous pharmacokinetic studies [19,20]. 26 Balb/c female mice were allocated into three group: G1 (n = 6): tumor control group, G2 (n = 10): low dose memantine group and G3 (n = 10): high dose memantine group. G1 was inoculated with 4T1 cells without any memantine treatment. G2 and G3 were pretreated with 5 and 10 mg/kg memantine daily intraperitoneal (ip) injection (weekend off) for 10 days, respectively. Both of G2 and G3 were subdivided into two groups as 2a (n = 4) and 3a (n = 4): tumor free groups and 2b (n = 6) and 3b (n = 6): tumor bearing groups. The design of in vivo experimental tumor model as groups were shown in Figure 1. 4T1 cells (5 × 10^4^/100 ul in Phosphate Buffered Saline (PBS) were inoculated subcutaneously into to left inguinal mammary tissue of the mouse for all group (G1, G2 and G3). Tumor volume was measured using caliper three times a week (for 10 days) starting from the 7th day of inoculation when the tumor became palpable. Tumor volumes were calculated by using the formula V= (W2 × L)/2, where V is tumor volume, W is tumor width, L is tumor length.

### 2.3. Tumor genomic DNA isolation and 5-methylcytosine (5-mC) level quantification

**Figure 2:**
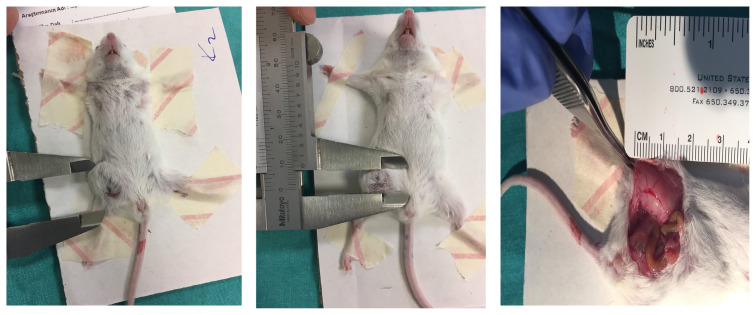
Representative images of 4T1 cell allograft tumors on inguinal region of Balb-c mice. Tumor volumes were calculated by using caliper.

Tumor length (L) and width (W) were measured by using the caliper. The image of tumoral masses on inguinal region were shown in Figure 2. The measurements recorded every other day and calculated according to the following formula: V = (W2 × L)/2. Tumor tissues were washed with phosphate buffered saline (PBS), and 1mm^3^ of each were transferred into Trizol isolation reagent (Invitrogen, Thermo Fisher Scientific, MA, USA). Tissues were disrupted by using the homogenizer (IKA, Germany) and centrifugated at 13.500 rpm at 4 ^o^C. Supernatants were transferred into another tubes. Genomic DNA isolation was performed according to the manufacturer’s instruction. DNA samples were quantified by NanoDrop ND-1000 (Thermo Fisher Scientific, MA, USA). 5-mC analysis was performed using the MethylFlash Methylated DNA Quantification kit (Epigentek, Farmingdale, NY, USA) according to the manufacturer’s instruction, and the absorbance was read at 450 nm using a microplate reader (Spectramax M3, Molecular Devices, USA).

### 2.4. Measurement of spleen interleukin 6 (IL-6) levels

Spleen tissue samples were homogenized and centrifuged at 13.500 rpm in Radioimmunoprecipitation assay (RIPA) lysis buffer. After the quantification of proteins with the Bicinchoninic acid (BCA) protein assay, IL-6 levels were quantified by using the Mouse Interleukin 6 (IL6) (ELISA Kit (Elabscience, Texas, USA) according to the manufacturer’s instructions. The absorbance was measured at 450 nm by Spectramax M3 microplate reader (Molecular Devices, Silicon Valley, California, USA).

### 2.5. Statistical analysis

Experiments were performed in technical triplicates. GraphPad Prism 8 was used in the statistical analysis. Tumor volumes were compared by using Friedman test, Dunn’s multiple comparison test when comparing 3 groups, and the p value was calculated as 0.0248 when comparing the control versus 10 mg treatment group. Spleen IL6 levels were compared by Friedman test, Dunn’s multiple comparison test. The genome wide methylation levels were compared by Šídák’s multiple comparisons test. Statistical analysis tables were represented in the corresponding results section. The value of p <0.05 was considered statistically significant.

## 3. Results

### 3.1. Memantine’s effect on tumor volume in the mouse 4T1 breast cancer model

**Figure 3:**
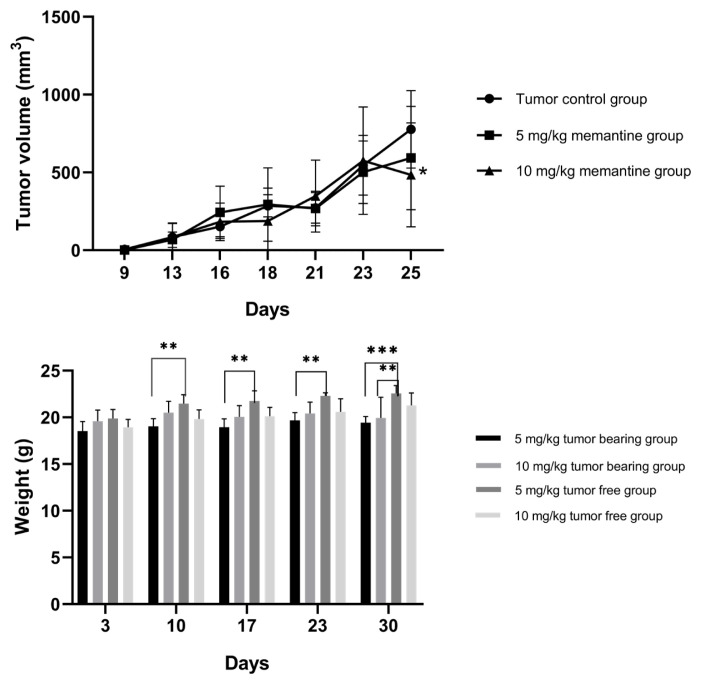
(A) Tumor volume measurement of G1 (n = 6): tumor control group, G2 (n = 10): Low dose (5 mg/kg) memantine group. G3 (n = 10): High dose (10 mg/kg) memantine group. 0 mg/kg memantine daily intraperitoneal (ip) injection for 10 days. Tumor growth was recorded for every 2–3 days. G3 decreased tumor volumes significantly (0.0248, Friedman test. (B) Effect of memantine treatment on the weights of G2 (n = 10): Low dose (5 mg/kg) memantine group, G2a (n = 4): Low dose (5 mg/kg) tumor free group, G2b (n = 6): Low dose (5 mg/kg) tumor bearing group, G3a (n = 4): High dose (10 mg/kg) tumor free group. G3b (n = 6): High dose (10 mg/kg) tumor bearing group.

In this study, memantine’s effect on tumor volume was investigated in 4T1 mouse breast cancer allograft model. Palpable tumors were first observed at 7 days after 4T1 cell inoculation in low (5 mg/kg) and high (10 mg/kg) dose tumor bearing groups (G2b and G3b), whereas it was observed after 9 days of cell inoculation in the low (5 mg/kg) (G2a) and the high (10 mg/kg) dose (G3a) tumor free groups for 10 days. As shown in Figure 3a, tumor volumes decreased in tumor free groups (G2a and G3a) when compared to tumor control group (G1). We found that tumor volumes decreased significantly (p = 0.0248) at high dose tumor free group (G3a) (Figure 3a). Tumor volume comparisons between the treatment groups were shown in Table 1.

Mice weights were also recorded during the whole memantine treatment protocol. The effect of memantine pretreatment on the weight of mice is shown in Figure 3b. We found that the weight of the low dose tumor bearing group (G2b) was significantly (p = 0,0014) reduced compared to the low dose tumor free group G2a (Figure 3b).

### 3.2. Spleen IL-6 levels were elevated in all groups

**Figure 4:**
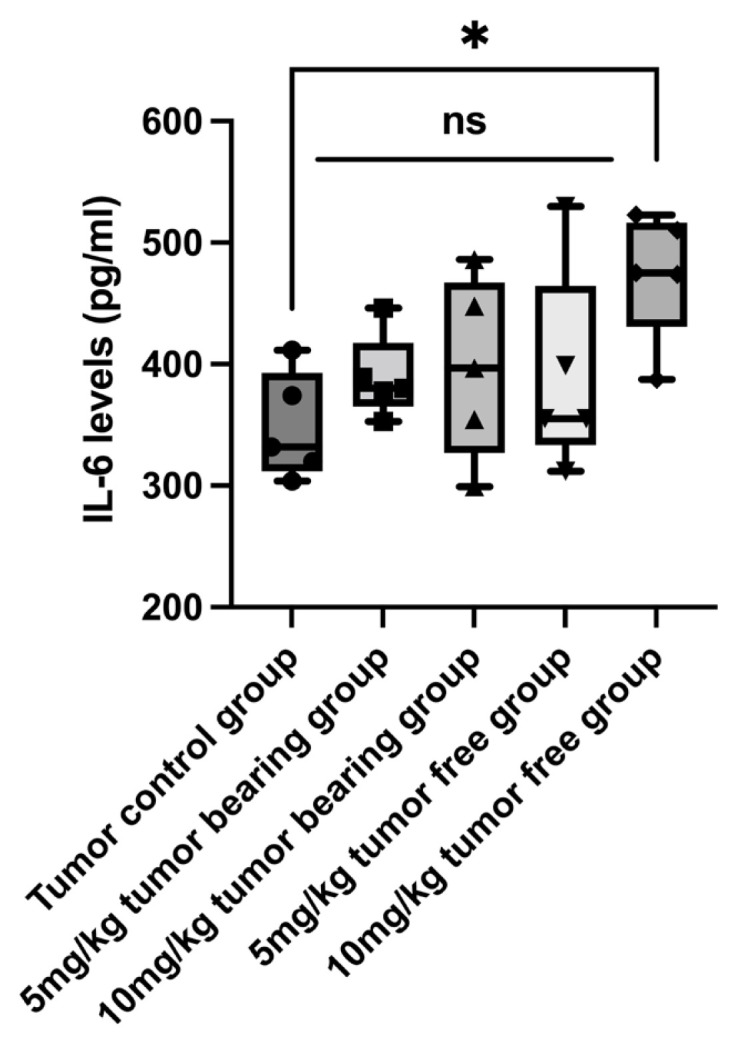
Spleen IL-6 level in G1 (n = 6): tumor control group, G2a (n = 4): Low dose (5 mg/kg) tumor free group, G2b (n = 6): Low dose (5 mg/kg) tumor bearing group, G3a (n = 4): High dose (10 mg/kg) tumor free group and G3b (n = 6): High dose (10 mg/kg) tumor bearing group. ns: not significant.

As memantine’s chemopreventive effect on breast cancer tumor volume has not been studied previously, we aimed to investigate its effect on immunogenicity as well. Therefore, we used IL-6 as a T cell response indicator to observe any immunological changes relevant to memantine pretreatment. As spleen is an indicator of the immunogenic response, we measured the spleen IL-6 levels. We found that spleen IL-6 levels were higher in low dose (5 mg/kg) (G2) and high dose (10 mg/kg) (G3) mematine groups when compared with tumor control group (G1) (p = 0.0204) (Figure 4, Table 2). Memantine increase the overall spleen IL-6 levels in all treatment groups (G2 and G3) that might indicate the T-cell induction.

### 3.3. Memantine treatment reduced 5-methylcytosine level in vivo

**Figure 5:**
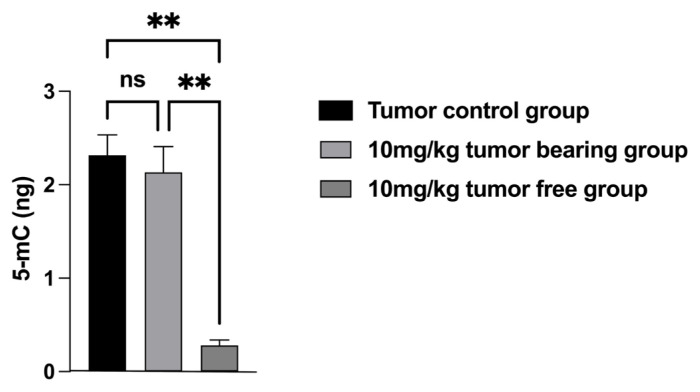
Effects of in vivo memantine treatment on tumor genomic DNA 5-methyl cytosine (5-mC) levels (ng) in G1 (n = 6): tumor control group, G3a (n = 4): High dose (10 mg/kg) tumor free group, G3b (n = 6): High dose (10 mg/kg) tumor bearing group. ns: not significant.

Global DNA methylation level change is important during cancer pathogenesis and progression in different types of cancer. Therefore, we wanted to evaluate tumor DNA methylation profiles in tumor control group (G1) and high dose (10mg/kg) memantine group (G3). We measured the effect of memantine treatment on global DNA methylation by using the colorimetric measurement levels of 5-methylcytosine (5-mC) in DNA samples. We evaluated 10 mg/kg memantine’s effect on 5-mC levels in vivo. We found that high dose tumor free group (G3a) has significantly lower genome-wide DNA methylation levels when compared to tumor control group (G1) p = 0.0012 and high dose tumor bearing group (G3b) p = 0.0020 (Figure 5, Table 3).

## 4. Discussion

Drug repurposing is defined as discovering the new uses for old drugs that is a new track for drug development. Repositioning approach accelerates the drug development process and also presents more available, cheaper and reliable drugs with known or fewer adverse effects [21]. The repositioned drugs have been successfully used for the treatment of most aggressive triple negative breast cancer. As drug repositioning is a fast tract process. It is becoming more attractive for the pharmaceutical industry.

The 4T1 mammary carcinoma can initiate spontaneously distant metastasize from the primary tumor in the mammary gland to multiple locations particularly to the lung [22]. Breast cancer is a heterogenous disease, responsible for the second cause of death by cancer. Therefore, unravelling the complexity of breast cancer biology remains crucial [23]. Tumor microenvironment changes, the methylation status of genes, and altered tumor metabolism gave rise to a new subset of cells that keep up the malign growth and spread [24]. In this study, we evaluated the chemopreventive effect of memantine in 4T1 mice breast cancer formation. We found that 10 days treatment of the low (5mg/kg) and high (10 mg/kg) dose memantine groups (G2 and G3, respectively) decreased the tumor volumes compared to tumor free groups (G2a and G3a). Tumor volumes were significantly (p = 0.02) lower at high dose memantine group (G3). Tumor free groups (G2a and G3a) did not lose weight. Memantine was well-tolerated, and we did not observe any behavioral abnormalities.

As memantine’s anticancer mechanism of action has not been studied in vivo, we aimed to investigate memantine’s effect on immunogenicity. Therefore, we used IL-6 as a T cell response indicator to observe any immunological changes after memantine treatment. IL-6 plays an important role in the acquired immune response by stimulating the production of effector T-cell development [25]. Moreover, IL-6 can induce proliferation or differentiation of many nonimmune cells [26]. Although IL6 signaling in tumor microenvironment might be considered as a bad player that promotes tumor progression, recent research shows that IL6 signaling mobilize T cell immune response to control tumor progression [27]. We found that memantine treatment triggered spleen IL-6 production that might evoke immune response in mice and could be the underlying reason for decreased tumor volumes. However deeper molecular analysis required in order to better understand the preventive mechanism of the drug.

DNA methylation is a major epigenetic change that plays critical role in the regulation of gene expression and is commonly deregulated in cancers [28]. However, that epigenetic change remains controversial in the literature as whether hypo or hypermethylation status of the genes contribute to the malignant transformation in cancer. A body of evidence also suggests that gene specific methylation changes might be more critical in the disease course [29]. In this study, we found that tumor control group (G1) has higher levels of genome wide methylation in their tumor DNA. High dose (10 mg/kg) memantine group (G3b) for 10 days was not effective in terms of reducing the methylation levels at the level of tumor control group (G1).

Our study is limited due to dosage-related concerns as memantine has very short half life (<4h) in mice compared to humans (60–80 h) [20]. However, we believe that our study will raise awareness and trigger new prospective studies related with its anticancer mechanism of action. “Old drugs with new tricks” approach is ongoing as most of the compounds in phase I trials fail in later stages of clinical development [30]. To the best of our knowledge, this is the first study to test memantine in animal models in cancer prevention setting. Therefore, we believe that this paper will give insights about the potential chemopreventive effect of memantine.

## Figures and Tables

**Table 1:** Tumor volume comparison between the treatment groups.

Dunn’s multiple comparisons test	p value according to tumor control group (G1) (n = 6)
Low dose (5mg/kg) memantine group (G2) (n = 10)	0.4226
High dose (10 mg/kg) memantine group (G3) (n = 10)	0.0248

**Table 2:** Spleen IL6 levels statistical analysis.

Dunn’s multiple comparisons test	p value according to tumor control group (G1) (n = 6)
Low dose (5 mg/kg) tumor free group (G2a) (n = 4)	>0.9999
High dose (10 mg/kg) tumor free group (G3a) (n = 4)	0.0204
Low dose (5 mg/kg) tumor bearing group (G2b) (n = 6)	>0.9999
High dose (10 mg/kg) tumor bearing group (G3b) (n = 6)	>0.9999

**Table 3:** Genome wide methylation level comparisons among the treatment groups. NS: not significant

Šídák’s multiple comparisons test	Summary	p value
High dose (10 mg/kg) tumor free group (G3a) (n = 4) according to tumor control group (G1) (n = 6)	^**^	0.0012
High dose (10 mg/kg) tumor bearing group G3b (n = 6) according to tumor control group (G1) (n = 6)	NS	0.9106
High dose (10 mg/kg) tumor bearing group G3b (n = 6) according to high dose (10 mg/kg) tumor free group G3a (n = 4)	^**^	0.0020
